# Periodicity of Intermittent Fever

**Published:** 1860-03

**Authors:** D. L. Robinson

**Affiliations:** New Elizabeth, Ind.


ARTICLE 5.

### PERIODICITY OF INTERMITTENT FEVER.

BY D. L. ROBINSON, M. D., OF NEW ELIZABETH, IND.

The periodicity of intermittent fever, has long been a theme of great interest among medical men. They have in all past ages exhausted their medical talent in trying to account for, and put forth a rational, and consistent theory for the mysterious, and regularly periodical return of the malady when it has once attacked its unfortunate victim. There is so much conflict among our best medical authorities on this subject, and so many inconsistent hypotheses, that I have almost concluded that the best of them knew but little about it. I am of the opinion that Miller in his *Principles of Medicine*, has come nearer the truth in the premises than any authority I have yet consulted. He says:—"One of the most remarkable characters in the disease resulting from malaria, is the periodicity of their attacks, and the diminution or cessation of the symptoms in the interval. This is probably due to the alternate accumulation of the malarious influence in the body and the reaction of the vital powers against it."

Now I propose in the present article to add my *mite* in as brief and concise a manner as possible, to the numerous *hypotheses* and *conjectures* that have been made and recorded with regard to this interesting subject—the periodicity of intermittents.

We are told—and rationally told too—that a bony tumor growing upon the inner tablet of the skull bone, and dipping down into the brain structure, gives rise to a very high degree of irritation; and that, that irritation causes the most alarming epileptic convulsions—that those convulsions are paroxysmal, having complete intermissions of perfect quietude between the paroxysms. Now the important question very naturally arises just here, why are those convulsions paroxysmal—the cause is continuous—present all the while? Our medical authors very consistently solve this problem for us. They say that this constant source of irritation in the brain, is continually giving rise to excitement of brain and nervous system; that this excitement (or irritation) accumulates to a sufficiently high degree to cause a convulsion; the convulsion persists until excitability in the brain and nervous system is exhausted; then a stasis, or period of comparative quietude, longer or shorter, is observed, until the susceptibility to excitement from this source of irritation present, is recovered, at which time, excitement of brain and nervous system again accumulates to a sufficient degree to cause another convulsion or paroxysm.

It will be easy to observe the end aimed at by this illustration. It is to put forth the idea—however absurd it may seem—that the cause of irritation (or *sedation* if you please) in an intermittent, acts upon the *same principle* in giving rise to paroxysms of ague, that the bony tumor or spicula of bone does in epileptic convulsions.

The *cause* of intermittent fever, all agree, is *marsh miasmata*; and I contend that its principle process of reaching the human system is through the medium of the atmosphere; that it is inhaled into the lungs, thereby admitted into the circulation in general, and diffused throughout the entire system. It first blunts the sensibility, or paralyzes the extremities, or periphery of the whole nervous system. Thus it suspends nervous innervation to the superficial, or capillary circulation in general, causing the blood to recede to the internal viscera, the spleen, liver, and vital organs generally; thereby diminishing animal heat *externally*, and causing congestion *internally*, and *constituting* what is termed the cold stage, or chill. This stage, or condition, persists until the nervous system survives the first impression, or shock, produced by the malarial poison; then those overburdened organs make an effort to relieve themselves of their ponderous load of blood; by that means, *reaction*, the hot stage, or fever, is developed. This fever may persist for a longer or shorter period, being governed by the amount of poison present, the temperament and condition of the individual; at all events, it persists until excitability in the nervous system is exhausted, at which time we have the apyrexia, or intermission, which lasts until the nervous system regains susceptibilty to the exciting influence of the miasmatic poison; then it sets up its irritation again, and the system is brought under the same excitement again, which constitutes another paroxysm, or *fit of ague*. Thus it continues to operate until nature, by the paroxysms, eliminates the whole of the miasmatic poison, or until it is neutralized by remedial agents.

The paroxysms may assume either the quotidian, tertian, or quartan type; that being governed by the amount of the poison diffused in the system, the constitutional vigor, and the temperament of the individual afflicted. Thus, the more constitutional vigor a person of *nervo-sanguine* temperament may have, and the more of the miasm there is in the system, the severer the attack will be, and the sooner the patient will rally from one paroxysm, and go into another. While on the contrary, an individual of the *bilio-lymphatic* temperament who has the system but moderately impregnated with the malaria, will have milder attacks of ague, the paroxysms be longer in passing off, and a longer period between paroxysms.

Enough has been said to set forth my theory upon the periodicity of intermittents, and I here rest the subject, with a hope that also enough has been said to call forth the opinion of the medical brethren generally, upon this very interesting subject.

---

Arsenious Acid in Chorea.—M. Barthez gives a detailed account of the case of a girl, eight years old, who had been quite healthy previously, but, in consequence of a sudden fright, had been attacked with chorea, six weeks before coming under his care. The patient recovered in a week, under the use of about one-twelfth to one sixth of a grain of arsenious acid three times daily.—*Gazette des Hopit.*

